# Portal Hypertension and Myeloproliferative Neoplasms: A Relationship Revealed

**DOI:** 10.1155/2013/673781

**Published:** 2013-09-16

**Authors:** Ahmet Burak Toros, Serkan Gokcay, Guven Cetin, Muhlis Cem Ar, Yesim Karagoz, Besir Kesici

**Affiliations:** ^1^Liv Ulus Hospital, Department of Gastroenterohepatology, 34347 Istanbul, Turkey; ^2^Istanbul Education and Research Hospital, Department of Internal Diseases, 34098 Istanbul, Turkey; ^3^Bezmialem University Medical Faculty, Department of Haematology, 34093 Istanbul, Turkey; ^4^Istanbul Education and Research Hospital, Department of Haematology, 34098 Istanbul, Turkey; ^5^Istanbul Education and Research Hospital, Department of Radiology, 34098 Istanbul, Turkey; ^6^Istanbul Education and Research Hospital, Department of Gastroenterohepatology, 34098 Istanbul, Turkey

## Abstract

*Background/Objectives.* Patients with myeloproliferative neoplasms have a well-established increased risk of thrombosis. Many trials report identification of an underlying myeloproliferative neoplasm by investigation of the patients developing portal hypertensive esophagus and/or fundus variceal hemorrhage in the absence of any known etiology. This trial was designed to investigate the association between myeloproliferative neoplasms and portal hypertension and to detect the frequency of portal hypertension development in this subset of patients. *Methodology.* Twenty-nine patients previously diagnosed with polycythemia vera, essential thrombocytopenia, and primary myelofibrosis, who were under followup at the hematology outpatient clinic of our hospital, were included in the trial. *Results.* In our trial, we detected portal hypertension in 13.8% of the patients (*n* = 4), as a finding that was similar to those obtained in other studies performed to date. *Conclusions.* Considering the fact that diagnosis of myeloproliferative neoplasms usually takes a long time, treatment should be started (while, on the other hand, assessing the investigational and therapeutical choices for the complications) right after the bone marrow biopsy or cytogenetic studies required for establishing the final diagnosis have been performed.

## 1. Introduction

The myeloproliferative neoplasms (MPN) include the Ph- (Philadelphia-) chromosome-positive chronic myeloid leukemia (CML), the Ph-chromosome-negative primary myelofibrosis (PMF), the polycythemia vera (PV), and the essential thrombocythemia (ET) [1–3]. Myeloproliferative neoplasms involve increased neoplastic proliferation in one or more series of the myeloid cells. In cases of polycythemia, overproduction of erythrocytes occurs. The increased erythrocyte mass leads to an increased blood viscosity. 

For PMF, extramedullary hematopoiesis (myeloid metaplasia) with the presence of myeloid, erythroid, and megakaryocytic series in locations other than the bone marrow such as the spleen, the liver, and the lymph nodes and fibrosis of various grades in the bone marrow suggest that the disease results from the hematopoietic stem cells [4–6]. Patients with PMF may develop severe portal hypertension, ascites, esophagus and fundus varices, intraluminal hemorrhage, and hepatic encephalopathy as a result of the massively increased splenoportal blood flow and the decreased hepatic vascular compliance or the hepatic venous thrombosis [7–9].

In essential thrombocythemia, an increase in the circulatory platelets occurs as a result of the continuous proliferation of megakaryocytes. The disease is typically characterized by splenomegaly and megakaryocyte hyperplasia in the bone marrow. Approximately 50% of the patients have JAK2 mutation. Thrombotic complications represent the most significant reason for morbidity and mortality among ET patients. 

While myeloproliferative neoplasms are referred to as separate entities, they share certain features due to their pathophysiologic bases. These include normal or high platelet count and platelet dysfunction. Platelet dysfunction may result in thrombosis. These thromboses may involve various sites of the body and may particularly be located in regions of vital importance such as the deep calf veins, the abdominal veins or arteries, the pulmonary veins or arteries, and the intracerebral veins or arteries. Patients may present with various manifestations including deep vein thrombosis, pulmonary hypertension, cerebral ischemia, portal-vein-thrombosis-associated portal hypertension, and secondary complications. 

Portal hypertension usually occurs following obstruction of the portal blood flow at any point throughout its course; the most common cause is the liver cirrhosis [10–12]. 

Portal hypertension is the main complication of the liver cirrhosis and is responsible for many complications including esophagus and/or fundus variceal hemorrhage, ascites, and encephalopathy [13]. Normally, portal venous pressure is less than 5 mmHg. If the resistance to the blood flow in the portal-hepatic vascular bed is increased, portal venous pressure will increase. When the portal pressure exceeds 10–12 mmHg, collaterals start draining the blood from the high-pressure portal vascular system to the low-pressure systemic venous circulation [10].

Portal hypertension may also manifest as a complication of the myeloproliferative neoplasm. This trial was designed to investigate the association between myeloproliferative neoplasms and portal hypertension development. Thus, we may have the opportunity to take early measures. 

## 2. Materials and Method

Patients above 18 years of age with Ph-chromosome-negative MPN, that is, with diagnosis of PV, ET, or PMF, who were under followup at the Istanbul Education and Research Hospital, were enrolled in the trial. Patients with previous portal hypertension secondary to causes other than MPN were excluded from the trial. The trial was approved by the local ethics committee, and informed consent was obtained from the participants. Patients selected among those followed up at our hematology clinic were referred to the gastroenterology outpatient clinic to undergo biochemical tests, whole blood counts, investigation of coagulation parameters, viral hepatitis markers, autoimmune hepatitis markers, serum ceruloplasmin levels, and esophagogastroduodenoscopy and portal Doppler examinations. Endoscopies and portal Doppler ultrasonographic investigations were performed by a gastroenterologist and a radiologist employed at our hospital, respectively. 

The “congestion index” is used to mean the ratio between the cross-sectional area (cm^2^) and the blood flow velocity (cm/sec) of the portal vein, as determined by a duplex Doppler system. It is suggested that the congestion index reflects the pathophysiological hemodynamics of the portal venous system in portal hypertension. Hepatic congestion index = portal vein sectional area (cm^2^)/mean blood flow velocity (cm/sec). Normal range is between 0.07 and 0.09 cm × sec [14].

The upper limit for hepatomegaly was 140 mm, and the upper limit for splenomegaly was 120 mm.

NCSS (Number Cruncher Statistical System) 2007&PASS (Power Analysis and Sample Size) 2008 Statistical Software (UT, USA) was used for performing the statistical analyses. For assessment of the trial data, the Mann-Whitney *U* test was used in comparing the data and the descriptive statistical methods (mean, standard deviation, median, frequency, and ratio). For comparison of the qualitative data, Fischer's exact test was used. The significance was set at a level of *P* < 0.05.

## 3. Results

The trial was conducted in a total of 29 cases. All of the patients enrolled were Ph-chromosome-negative PV, ET, and PMF patients, previously diagnosed clearly by peripheral blood-sample-bone-marrow investigation and cytogenetic analyses. 

All of the 29 patients had biochemical values and whole blood counts within normal limits. Markers were negative for B and C viruses. Serum ceruloplasmin, serum iron, and total iron binding capacity were investigated and were found to be normal. While some abnormalities were detected in autoimmune hepatitis markers, the presence of normal biochemical values and coagulation parameters indicated the absence of an active autoimmune hepatitis.

The age range was 18–78 years with a mean patient age of 54, 0 ± 16, 37 years. The duration of diagnosis varied between 2 and 7 years with a mean duration of 3, 24 ± 1, 27 years; 41.4% (*n* = 12) of the cases were males, and 58.6% of the cases were females (*n* = 17). 

69% of the cases (*n* = 20) had essential thrombocythemia, 6.9% had (*n* = 2) primary myelofibrosis, and 24.1% (*n* = 7) had PV diagnosis. 

One of the patients was detected to have portal vein thrombosis, and the other one was detected to have splenic vein thrombosis; therefore, portal vein and splenic vein diameters could not be measured in those patients, but those patients were diagnosed with portal hypertension. In one patient, the splenic vein diameter and the spleen size could not be measured since the patient had previously undergone splenectomy. The presence of a congestion index above 0.09 cm × sec was considered to indicate portal hypertension. 75.9% of the patients had hepatomegaly (*n* = 22), and 44.8% had (*n* = 13) splenomegaly (Table 1). 

6.9% of the cases (*n* = 2) had esophagus and/or fundus varices, while 13.8% (*n* = 4) had portal hypertension. The direction of the flow is hepatopedal in 96.6% of the patients. In one patient, no flow could be detected due to thrombosis (Table 2). 

There was no statistically significant difference between the different types of disease in portal hypertension (*P* > 0.05). Basically, portal hypertension may occur in all of them and is not specific to the disease (Table 3). 

## 4. Discussion

PV is a slow-course chronic disease. Thrombotic episodes are quite common, and for 1/3 of the patients, they are the most significant complications of PV [15]. Thromboses may be venous or arterial. Excessive erythrocytosis and an increase in blood viscosity, thrombocytosis, and platelet dysfunction primarily account for the thrombotic events. Portal hypertension, esophagus and/or fundus varices, and abdominal pain are not common in PV patients and occur due to splenic and hepatic vein thromboses [16]. 

In PMF patients, both the arterial and the venous thromboses risks are increased; but these risks are not as high as those of PV and essential thrombocythemia [17]. Patients with PMF may develop severe portal hypertension, ascites, esophagus and fundus varices, intraluminal hemorrhage, and hepatic encephalopathy as a result of the massively increased splenoportal blood flow and the decreased hepatic vascular compliance or hepatic venous thrombosis [7–9]. Thrombotic complications represent the most significant reason for morbidity and mortality among ET patients [18, 19]. 

Irrespective of the cause, all forms of PH are characterized by splenomegaly in the presence or absence of esophageal variceal vein-associated symptoms and hypersplenism [20]. In cases of portal vein thrombosis, ascites or jaundice is not observed since the liver is normal. In case of hepatic vein occlusion, findings of various grades of hepatic failure and ascites are almost always observed. 

The detection of thrombosis in intra-abdominal large veins of 2 patients diagnosed with portal hypertension but the failure to detect such a mechanism in the other 2 patients suggested that not only the large vein thromboses but also the small undetected thromboses could represent a risk for portal hypertension. 

Similarly, noncirrhotic portal hypertension (NCPH) may be a reason, which is described as follows: a poorly understood condition characterized by the presence of portal hypertension in the absence of conventional hepatic cirrhosis (defined as diffuse nodules totally surrounded by fibrous septa). It has been described in association with blood coagulation disorders and myeloproliferative neoplasms, with exposure to toxic substances or to drugs and with immunological diseases. Most of the cases present with thrombosis or sclerosis of the intrahepatic portal venous system with variable involvement of the prehepatic portal system [21].

In this trial, we investigated the incidence of portal hypertension occurrence and the development of esophagus and/or fundus varices in Ph-chromosome-negative patients, who have been diagnosed with myeloproliferative neoplasms. 

 The review of similar trials in the literature revealed that the basic hematologic diagnosis was established after development of fatal portal hypertension complications such as esophageal variceal hemorrhage in some patients [22, 23]. We also observed that an incidence of portal hypertension between 10% and 17% was detected among patients with myelofibrosis [24–31]. In essential thrombocythemia, the thrombosis risk is increased up to 24% following a 27-month treatment-free period [32]. Therefore, one in each 4 patients should be expected to develop thrombotic complications after 2 years. 

In our trial, 69% of the cases (*n* = 20) had essential thrombocythemia, 6.9% had (*n* = 2) primary myelofibrosis, and 24.1% (*n* = 7) had PV diagnosis. The biochemical values were within the normal limits. Markers were negative for B and C viruses. Serum ceruloplasmin, serum iron, total iron binding capacity were investigated, and metabolic liver diseases such as hemochromatosis or Wilson disease were ruled out. While some abnormalities were detected in autoimmune hepatitis markers, the presence of normal biochemical values and coagulation parameters indicated the absence of an active autoimmune hepatitis. We excluded patients with a known hepatic disease because we wanted to know the rate of portal hypertension arising solely from myeloproliferative neoplasms.

Doppler ultrasonographic examination detected portal vein thrombosis in one patient and splenic vein thrombosis in another. This condition, which the patients were not previously aware of, shows that this group of patients may also have silent thrombosis. 

Of the 29 patients participating in our study, 16 were positive for JAK2 mutation. Two of the four patients with PH were positive for JAK2; and those were the ones with portal and splenic vein thromboses.

Splenomegaly and hepatomegaly occur as expected findings in the course of portal hypertension and myeloproliferative neoplasms both as a result of extramedullary hematopoiesis and excessive breakdown. 75.9% of the patients had hepatomegaly (*n* = 22), and 44.8% had (*n* = 13) splenomegaly. Because we have already excluded chronic hepatic diseases of any form, hepatomegaly found in the study group is of hematologic origin, with only increasing dimensions and without nodule formations or biochemical abnormalities of cirrhosis. 

Esophagogastroscopy performed in 2 patients revealed esophagus and/or fundus varices, while 4 patients were detected to have abnormalities by Doppler ultrasonography. Patients with esophageal varices and those with a congestion index above the normal value of 0.09 as detected by portal Doppler ultrasonography were considered to have portal hypertension. Anamnesis of those patients did not include alcohol addiction, and their laboratory values were normal; therefore, we thought that the cause of PH was MPN in those patients. 

We detected 4 patients with PH in our trial. The ratio of those patients (*n* = 4) to the total number of patients (*n* = 29) was 13.8%. The literature reports reveal similar rates (10%–17%) for essential thrombocythemia, PV, and PMF [24–31]. 

However, due to the fact that these are rare diseases, inadequate number of patients is a drawback. No relation was found between the types of MPN and PH generation; similarly, no relation was shown with the disease duration and the generation of PH. The main reason for that is the small number of patient groups. For example, the small number of PMF cases falsely leads to the result that this group had a high rate of portal hypertension. The absence of a balanced distribution between the patient groups investigated makes an intergroup comparison inappropriate. 

The patient can experience a complication such as portal hypertension as from the start of the disease, and we should be careful in this respect. Accordingly, considering the fact that some patients may have been diagnosed late, we concluded that the investigation for complications should start with the establishment of diagnosis by bone marrow and cytogenetic analyses (esophagogastroscopy for esophageal and/or fundus varices and Doppler ultrasonography for silent portal hypertension or abdominal vein thrombosis). 

One should also keep in mind that thrombotic episodes could occur not only in intra-abdominal veins or arteries that could lead to portal hypertension but also in intracranial veins or arteries in a patient with headache or dizziness and in cardiac arteries in a patient with exertional or resting angina. 

## 5. Conclusion

In conclusion, the incidence of thrombotic episodes in Ph-chromosome-negative MPN patients was found similar to those reported in the literature. The presence of portal hypertension in 13.8% of the patients is remarkable. However, due to unbalanced distribution of patient groups, it would not be appropriate to make any comparison between the groups with respect to portal hypertension incidence. 

Occurrence of a complication such as portal hypertension appears to be usual in the course of these diseases. Precautions should be taken against thrombotic episodes before they lead to fatal complications. 

These complications can be experienced anytime. So, search for a complication should immediately be initiated once the hematological diagnosis is established. Knowing that thrombosis can occur not only in intra-abdominal arteries and veins but also in other regions, clinicians should utilize the appropriate diagnostic tools. 

## Figures and Tables

**Table 1:** Results of Doppler ultrasonographic examination.

	Min–Max	Med ± SD
Portal vein diameter (mm)	**10–12**	10.27 ± 2.92
Splenic vein diameter (mm)	**4–10**	7.33 ± 2.75
Congestion index (cm × sec)	0.002–2.26	0.11 ± 0.43

Hepatomegaly–*n* (%)	(present) 22 (75.9%)	(absent) 7 (24.1%)
Splenomegaly–*n* (%)	(present) 13 (44.8%)	(absent) 15 (51.7%)

**Table 2:** Frequency of esophageal (+/−) fundus varices and portal hypertension.

	Present	Absent
	*n* (%)	*n* (%)
Esophageal (+/−) fundus varices	2 (6.9%)	25 (92.6%)
Portal hypertension	4 (13.8%)	25 (86.2%)
Direction of flow (hepatopedal)	28 (96.6%)	1 (3.4%)

**Table 3:** Portal hypertension and disease relationship.

	Portal HT		Total, *n* (%)	*P*
	Present, *n*	Absent, *n*		
Essential thrombocythemia	1	19	**20 (69%)**	**0.076**
Primary myelofibrosis	1	1	**2 (6.9%)**	**0.261**
Policythemia vera	2	5	**7 (24.1%)**	**0.238**

Total	4 (13.8%)	25 (86.2%)	29 (100%)	

Fisher's exact test.
